# Altered electrochemical properties of iron oxide nanoparticles by carbon enhance molecular biocompatibility through discrepant atomic interaction

**DOI:** 10.1016/j.mtbio.2021.100131

**Published:** 2021-09-04

**Authors:** S.K. Verma, A. Thirumurugan, P.K. Panda, P. Patel, A. Nandi, E. Jha, K. Prabakaran, R. Udayabhaskar, R.V. Mangalaraja, Y.K. Mishra, A. Akbari-Fakhrabadi, M.J. Morel, M. Suar, R. Ahuja

**Affiliations:** a School of Biotechnology, KIIT University, Bhubaneswar, 751024, India; b Condensed Matter Theory Group, Materials Theory Division, Department of Physics and Astronomy, Uppsala University, Box 516, SE-75120, Uppsala, Sweden; c Advanced Materials Laboratory, Department of Mechanical Engineering, University of Chile, Santiago, Chile; d SRM Research Institute, SRM Institute of Science and Technology, Kattankulathur, Chennai, Tamil Nadu, 603203, India; e Advanced Ceramics and Nanotechnology Laboratory, Department of Materials Engineering, Faculty of Engineering, University of Concepción, Concepción, 4070409, Chile; f Technological Development Unit (UDT), University of Concepcion, Coronel Industrial Park, Coronel, Chile; g Smart Materials, NanoSYD, Mads Clausen Institute, NanoSYD, University of Southern Denmark, Alsion 2, Denmark; h Department of Physics, Indian Institute of Technology Ropar, Rupnagar 140001, Punjab, India; i Instituto de Investigaciónes Científicas y Tecnológicas (IDICTEC), Universidad de Atacama, Copayapu 485, Copiapó, Chile

**Keywords:** Magnetic nanoparticles, Super capacitors, Toxicity, Zebrafish, Oxidative stress, Apoptosis

## Abstract

Recent advancement in nanotechnology seeks exploration of new techniques for improvement in the molecular, chemical, and biological properties of nanoparticles. In this study, carbon modification of octahedral-shaped magnetic nanoparticles (MNPs) was done using two-step chemical processes with sucrose as a carbon source for improvement in their electrochemical application and higher molecular biocompatibility. X-ray diffraction analysis and electron microscopy confirmed the alteration in single-phase octahedral morphology and carbon attachment in Fe_3_O_4_ structure. The magnetization saturation and BET surface area for Fe_3_O_4_, Fe_3_O_4_/C, and α-Fe_2_O_3_/C were measured as 90, 86, and 27 emu/g and 16, 56, and 89 m^2^/g with an average pore size less than 7 nm. Cyclic voltammogram and galvanostatic charge/discharge studies showed the highest specific capacitance of carbon-modified Fe_3_O_4_ and α-Fe_2_O_3_ as 213 F/g and 192 F/g. The *in vivo* biological effect of altered physicochemical properties of Fe_3_O_4_ and α-Fe_2_O_3_ was assessed at the cellular and molecular level with embryonic zebrafish. Mechanistic *in vivo* toxicity analysis showed a reduction in oxidative stress in carbon-modified α-Fe_2_O_3_ exposed zebrafish embryos compared to Fe_3_O_4_ due to despaired influential atomic interaction with sod1 protein along with significant less morphological abnormalities and apoptosis. The study provided insight into improving the characteristic of MNPs for electrochemical application and higher biological biocompatibility.

## Highlights

• Carbon modification of octahedral shaped magnetic nanoparticles (MNPs) was done using sucrose.
• The magnetization saturation and BET for Fe_3_O_4_, Fe_3_O_4_/C and α-Fe_2_O_3_/C was 90, 86, 27 emu/g and 16, 56, 89 m^2^/g.
• Carbon modified α-Fe_2_O_3_ induce less ROS in exposed zebrafish embryos.
• Carbon modified Fe_3_O_4_ despair influential atomic interaction with zebrafish Sod1 protein.
• Carbon modified Fe_3_O_4_ exhibit less morphological abnormalities and apoptosis.

## Graphical abstract

**Figure:**
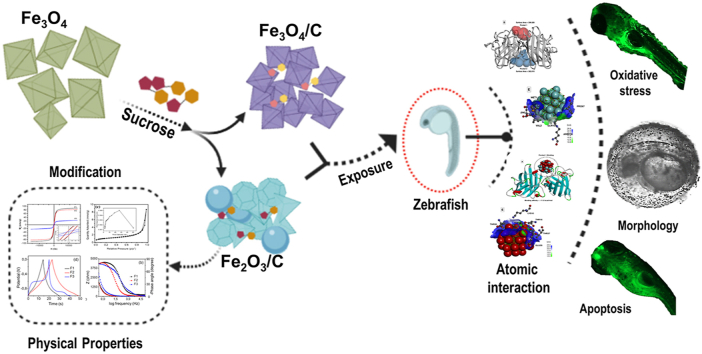

## 1 Introduction

The recent era has seen the application of nanomaterials in multidirectional technology whether it be new gadget, health equipment, drugs, or any new environmental and biomedical technologies [1,2]. The use of nanomaterials has a new dimension in these interdisciplinary applications. The nanomaterials designed by different chemical and physical engineering methods are used for biomedical purposes such as in the fabrication of biosensors, lab-on-chip devices, and so on. Electrochemical supercapacitors are one such device that has fascinated researchers for energy applications. They have been recognized due to several advantageous properties over the batteries such as higher power density, cycle lifetime, and opportunity to merge the energy and power gap between the conventional capacitors and batteries [[3], [4], [5]]. A larger number of both theoretical and experimental reports on the supercapacitor provide new insights on their applications [[6], [7], [8], [9]]. The suitable selection of electrode and electrolyte has been attempted by using several new materials and still progressing to overcome the challenges [10,11]. Magnetic nanoparticles (MNPs) such as iron oxide, cobalt oxide, and nickel-based nanomaterials have been investigated for the supercapacitor designing purpose. These nanoparticles have been reported to be deployed for many physical and environmental applications [12]. However, rigorous investigations at the laboratory scale and the use of the nanomaterials at the industrial scale have raised concern over their after-usage accumulation in the environment followed by their toxicological impacts. Hence, the need for eco-compatible materials having high productivity for supercapacitor application has urged.

An enormous amount of research effort has been put to improve the electrochemical properties of supercapacitor materials. The major effort has been put in improving the capacitance and the cycle stability to select the appropriate electrode materials in supercapacitors with an electrical double layer, pseudo-capacitive, and their combination materials [13,14]. The required properties such as larger surface area and high conductivity have been achieved by combining electrostatic and polymer conducting materials [15,16]. The selection of metal oxide as a pseudocapacitor (PC) electrode material mainly depends on the electronic conductivity and the different oxidation states within the compound. Researchers have reported the successful use of Ru oxide-based materials, Mn oxide-based compounds, Co_3_O_4_, V oxide-based materials, and NiO-based materials for the corresponding applications. However, these materials have limitations such as low theoretical capacitance value 1,100–1,300 F/g in case of Ru-based materials [17], low chemical reactivity in case of Mn-based compounds [5,18], and a high theoretical capacitance of 3,750 F/g and 2,854 F/g in V oxide [17] and NiO-based compounds [19]. Fe oxide-based compounds such as Fe_3_O_4_ and α-Fe_2_O_4_ have been found as suitable for PC electrode materials due to the higher theoretical capacitance value as 2,606 and 3,625 F/g [11]. The problem of having lower conductivity in Fe_3_O_4_ was solved by combining it with electric double layer capacitor (EDLC) materials. As a result, the capacitive characteristics were found enhanced by combining Fe_3_O_4_ and α-Fe_2_O_3_.

A part from iron oxide-based materials, carbon-based materials have also been recognized as suitable materials to prepare the hybrid electrode material (with various metal oxides) due to its easy preparation process, lower cost, larger surface area, and good electronic conductivity [20,21]. Carbon-modified Fe-based oxides have been found to be suitable for variable applications such as lithium-ion batteries and electrocatalyst. However, achieving various morphologies, enhancement in surface area and the specific capacitance for the Fe oxide materials are still a challenging process. Addressing the issue, we report here the development of hybrid magnetic nanomaterial by combining Fe oxide with carbon material.

Concerning the modern scenario of environmental sustainability, the eco-compatibility of any new nanomaterials has been a crucial factor for its applicability. The utility of the material during the usage as well as after-usage has to be considered in a limited way, provided their extent of effects on environment and biological identities. The deployed nanomaterials especially MNPs like iron oxide can be accumulated in the environmental sources after being used. These accumulated nanomaterials can further leak on abiotic resources like water, soil and show their impact on aquatic animals affecting the aquatic ecosystem. Hence, it is important to investigate the eco-compatibility of new synthesized material. Fe_3_O_4_-based nanoparticles have been reported for their toxicological effects on different cell lines [22] as well as mouse [23] and zebrafish model [24]. Carbon-based materials have also been reported for their eco-toxicological effects; however, they have also been found to be eco-compatible at a specific concentration and dosage [25]. Hence, it can be hypothesized that the proposed carbon-modified Fe_3_O_4_-based nanoparticles will have a synergistic and reduced toxicological impact on biological entities due to alteration in electrochemical properties of Fe_3_O_4_ by carbon modification. The hypothesis has been investigated in this report by evaluation of biocompatibility of synthesized nanomaterial with embryonic zebrafish model [26]. Moreover, the mechanism has also been investigated for better understanding of the biocompatibility at molecular and atomic level.

## 2 Experimental

### 2.1 Synthesis of Fe_3_O_4_ magnetic nanoparticles and carbon modification

Fe_3_O_4_ MNPs were prepared by alkaline precipitation (chemical oxidation method) [27]. In brief, 0.1 M of FeCl_2_.4H_2_O (3.976 g) was dissolved in double distilled water and stirred at 400 rpm using magnetic stirrer. At 80 °C, NaOH (0.2 M (1.6 g)) solution was added drop wise for the reaction process and then KNO_3_ solution was added for the oxidation process. NaOH was used for the metal hydroxide precipitation and KNO_3_ was used to oxidize the ferrous ions to the ferric state. The reaction was done in 200 mL of double distilled water. The solution was stirred at 90 °C for 2 h. The precipitates were collected using magnetic separation process and washed several times to remove residual unreacted products. The collected black powders were dried at 90 °C in the hot air over for 12 h.

For the carbon modification, 1 g of sucrose was mixed with double distilled water and stirred continuously until to get the clear solution. Then, 1 g prepared Fe_3_O_4_ MNPs were dispersed in the sucrose solution and sonicated to achieve the uniform dispersion of nanoparticles. The dispersion was further heated to 90 °C to convert the solution into semisolid. Furthermore, the mixture was heated to 200 °C for 12 h for the pyrolysis. The mixture was further heated at 600 °C and 800 °C for 2 h in nitrogen atmosphere. Followed by heating and cooling, the carbon-modified MNPs were washed several times with double distilled water and collected using magnetic separation process to remove excess carbon nanostructures unattached with MNPs. Final powders were dried at 80 °C for 4 h and named as F1, F2, and F3 corresponding to bare Fe_3_O_4_, carbon modified with 600 °C and 800 °C.

### 2.2 Physicochemical characterization

The synthesized MNPs were analyzed by X-ray diffraction (XRD) by Bruker D8 X-Ray diffractometer with a Cu K source. The morphology analysis of the MNPs was done with field emission scanning electron microscopy (FESEM) (Carl Zeiss Neon 40) and transmission electron microscopy (TEM; Tecnai F20 FEG at 200 kV accelerating voltage). Room temperature magnetic properties were analyzed by using vibrating sample magnetometer (VSM-Model 7404, Lakeshore, MI). All the electrochemical measurements were carried out by using CHI760E bipotentiostat (CH Instruments, Inc.) with KOH (6 M) solution. Ten milligram of prepared particles was mixed in 5 mL of stock solution (containing 20 mL isopropyl alcohol (IPA), 79.6 mL Milli-Q water, and 0.4 mL of Nafion) by sonication for 2 h. Solution (5 μL) was coated on a cleaned glassy carbon electrode (GCE) with an area of 0.0707 cm^2^ and kept at room temperature for slow evaporation. The cyclic voltammetry (CV) analysis was performed with a potential widow of −0.8 to 0.3 V vs. Ag/AgCl at various scan rate of 2–200 mV/s. A platinum (Pt) wire, Ag/AgCl (sat. KCl) electrode, and prepared composite-coated GCE were used as counter, reference, and working electrodes respectively. The specific capacitance was calculated from the galvanostatic charge/discharge at different current densities ranging from 0.5 to 20 A/g in the potential range of −0.1 to 0 V. The electrochemical impedance spectroscopy (EIS) analysis of the electrodes was studied with a perturbation of sinusoidal voltage signal of 10 mV applied over wide range of frequency between 1 MHz and 0.01 Hz with open circuit potential. The zeta potential was determined by using Zetasizer (Malvern, UK).

### 2.3 Zebrafish maintenance and embryo culture

All animal procedures were agreed by the relevant guidelines of Institutional Animal Ethics Committee (IAEC) of KIIT University. All experiments were done in accordance with relevant animal practice guidelines and regulations of IAEC, KIIT University. The adult zebrafish rearing and maintenance was achieved in an overflow container setup supplied by Aquaneering, USA. Equilibration of the system was done with fish water containing 75 g NaHCO_3_, 18 g sea salt, and 8.4 g CaSO_4_ per 1,000 mL. Nutritional regulation was done by providing feed three times in a day with blood worm containing fish food. The culturing and obtaining of embryos were done in breeding setup with net partition and breeding female:male in 2:1 ratio. Photoperiodism was maintained as per the standard regulation of 12 h dark and light respectively. Viable embryos were separated from non-viable ones after obtaining embryos and rinsing with the medium. All the experiments were performed in filter sterilized Holtfreter medium [28]. Chemicals used for preparing buffer were purchased from Merck.

### 2.4 Biocompatibility assessment

Comparative biocompatibility assessment of carbon-modified Fe_3_O_4_ and α-Fe_2_O_3_ was performed with 3- to 4-h post fertilized (hpf) embryos at blastula stage as mentioned by Verma et al. [29] and according to the OECD 236 guidelines. In brief, exposure of set of 20 embryos was done at different concentrations (0, 25, 50, 100 μg/mL) of Fe_3_O_4_ and α-Fe_2_O_3_/C for 72 h in 24-well plates with Holtfreter medium (HF) buffer. The experimental setup was incubated in 14/10 h light/dark at 28 ± 1 °C. Unexposed embryos were taken as control. The morphological changes were analyzed using stereomicroscope. Different morphological defects like notochord developmental defects, pericardial edema, and the swollen yolk were observed in the exposed embryos. Hatching rate was determined as ratio of embryos hatched by 72 h after fertilization compared to the untreated group. Mortality rate was evaluated as the percentage ratio of number of viable embryos compared to untreated one 72 h after fertilization. Heartbeat count per minute was taken to assess the heart rate. All the experiments were done in triplicates and repeated thrice.

*In vitro* biocompatibility assessment of Fe_3_O_4_ and α-Fe_2_O_3_/C was also performed with colon cell lines (HCT116) by evaluating the survivability of cells on exposure to Fe_3_O_4_ and α-Fe_2_O_3_/C. The survivability assay was performed by the 3-(4,5-dimethylthiazol-2-yl)-2,5diphenyltetrazolium bromide (MTT) assay as mentioned in Supplementary information [30].

### 2.5 Oxidative stress analysis

Oxidative stress analysis in Fe_3_O_4_ and α-Fe_2_O_3_/C exposed embryos was done by staining of treated and untreated embryos using 1.25 mg/L 2′,7′-dichlorofluorescin diacetate (H_2_DCFDA) dye for 20 min in dark [12]. The embryos were exposed as mentioned in protocol for biocompatibility assays. To extrapolate the extra stain, washing was performed with HF buffer. Analysis was performed using EVOS inverted fluorescent microscope (ThermoScientific). Data analysis and presentation was done with the help of ImageJ. All the experiments were performed in triplicates and statistical analysis was performed using GraphPad prism 6.

### 2.6 Real-time PCR analysis

The alteration in expression of sod1 protein in embryonic zebrafish due to exposure of Fe_3_O_4_ and α-Fe_2_O_3_/C was checked by real-time (RT) PCR analysis. To check the gene expression in zebrafish cells, cell suspension was obtained from the embryos treated with 25, 50, and 100 μg/mL for 72 h. The RNA was isolated using TRIzol reagent (Ambion, Foster City, CA) according to the manufacturer’s instructions. Consequently, RNase-free DNase I (Fermentas) treatment was done, and complementry dioxyribonucleic acid (cDNA) synthesis was performed using the Hi-cDNA Synthesis Kit (HIMEDIA, Mumbai, India). The analysis of RNA quantity and purity was done at each point by Nanodrop (Colibri Microvolume Spectrometer). Quantitative polymerase chain reaction (qPCR) was performed for each sample using the KAPA SYBR FAST qPCR Master Mix (2×) (Kapa Biosystems, Wilmington, MA) with a suitable cDNA dilution as a template. To normalize the tested genes’ expression (sod1), 16S (Housekeeping gene) was taken as an internal control. The determination of values of mean fold change expression values was done for three replicates. The primers used are listed in Table 1.

**Table 1:** Primers used for the RT-PCR analysis of mRNA expression zebrafish embryos exposed to Fe_3_O_4_ and α-Fe_2_O_3_/C nanoparticles.

Zebrafish	**Genes**	**Primers**	**Sequence (5'-3')**
	actB1	Forward	GCGTGCACTGAAAACTCACA
		Reverse	GCAACTAGCTTGAAACTCGCC
	sod1	Forward	ATTGAAATAGACGGTGCCGGT
		Reverse	CCTCATTGGTCGATTCCGCT

mRNA, messenger ribonucleic acid.

### 2.7 Assessment of apoptosis

Apoptosis analysis of exposed and unexposed embryos was done by Acridine orange staining as described by Verma et al. [12]. In brief, untreated and treated zebrafish embryos with Fe_3_O_4_ and α-Fe_2_O_3_/C were stained with 5 μg/mL acridine orange (AO) dissolved in HF for 20 min followed by washing with HF buffer for removal of extra stains. Images were taken using the green channel of EVOS inverted fluorescent microscope (ThermoScientific) for comparing the apoptosis that occurred in zebrafish embryos due to exposure of different concentration. Data analysis and presentation was done with the help of ImageJ. All the experiments were performed in triplicates and statistical analysis was performed using GraphPad prism 6.

### 2.8 *In silico* analysis

The protein sequence of zebrafish (*Danio rerio*) superoxide dismutase (sod1) has been retrieved from UniProt database bearing ID O73872 and subjected for homology modeling using SWISS-MODEL server. Active site pockets have been identified using CastP server. The α-Fe_2_O_3_ and Fe_3_O_4_ nanoparticles have been modeled using Crystal Maker software using the parameters, e.g., α-Fe_2_O_3_ unit cell with R 3̅ c space group and lattice parameters of a = b = 5.0380 Å and c = 13.7720 Å and Fe_3_O_4_ unit cell with F d 3̅ m space group and lattice parameters of a = b = c = 8.3941 Å. The modeled nanoparticles and the protein target were subjected for molecular docking using AutoDock Vina [31]. The docking after analyses were performed using Discovery Studio Visualizer and Chimera [32].

## 3 Results and discussion

**Fig. 1:**
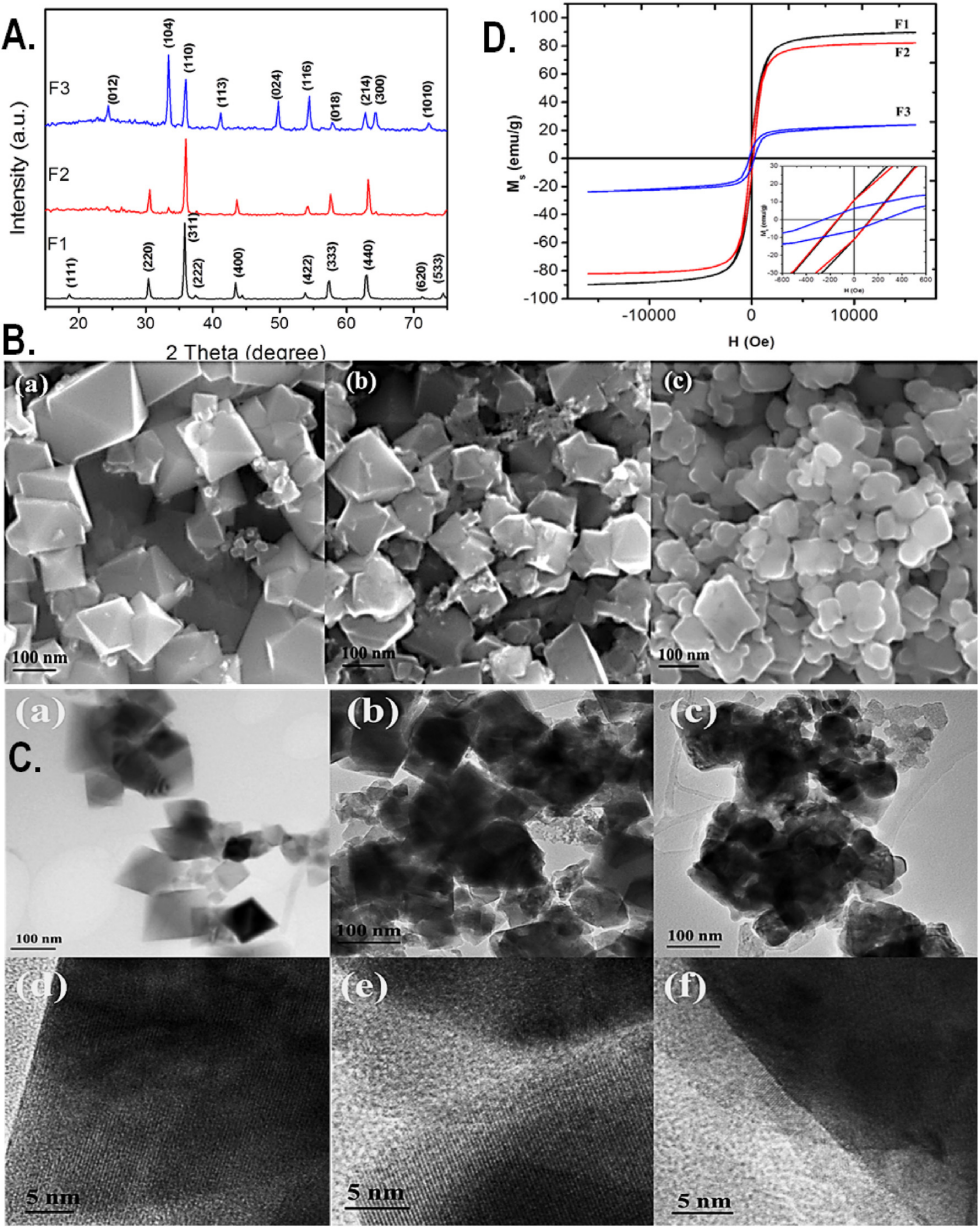
(A) XRD pattern of synthesized nanomaterials Fe_3_O_4_ (F1), Fe_3_O_4_/C (F2), and α-Fe_2_O_3_/C (F3) respectively. (B) FESEM micrograph of (a) Fe_3_O_4_, (b) Fe_3_O_4_/C, and (c) α-Fe_2_O_3_/C. (C) TEM and HRTEM micrograph of (a and d) Fe_3_O_4_, (b and e) Fe_3_O_4_/C, and (c and f) α-Fe_2_O_3_/C. (D) VSM hysteresis loop of synthesized nanomaterials. F1, F2, and F3 represent the graph corresponding to Fe_3_O_4_, Fe_3_O_4_/C, and α-Fe_2_O_3_/C.

The evolution of the materials for sustainable energy application demands study on materials with higher output and eco-compatibility. This study is intended toward the fabrication of an eco-compatible hybrid magnetic nanomaterial of Fe_3_O_4_/C and α-Fe_2_O_3_/C by modification of Fe_3_O_4_. The hybrid nanomaterials were synthesized through modification of Fe_3_O_4_ by carbon using sucrose as source material of carbon. The synthesized Fe_3_O_4_/C and α-Fe_2_O_3_/C material was then characterized for their physiochemical properties using standard physicochemical techniques. Fig. 1A shows the XRD pattern of Fe_3_O_4_, Fe_3_O_4_/C, and α-Fe_2_O_3_/C. Single phase was observed for the prepared bare Fe_3_O_4_ MNPs with an average grain size of 33 nm. The phase transition from Fe_3_O_4_ to α-Fe_2_O_3_ (JCPDS-89-8104) was observed for the samples calcined at 800 °C. Some minor peaks of α-Fe_2_O_3_ were observed for the sample calcined at 600 °C. The average grain size of Fe_3_O_4_ and α-Fe_2_O_3_ was estimated as 34 nm and 26 nm. The reduction in the grain size for α-Fe_2_O_3_/C is due to the smaller particle’s formation from the bigger particle during the phase transformation at higher calcination temperature. There were no characteristic reflections of any carbon allotropes in both the samples, which indicated that the carbon fraction might be less, or in amorphous form. Furthermore, to evaluate the morphology, the prepared samples were analyzed by the FESEM analysis. Fig. 1B shows the FESEM micrograph of bare and carbon-modified Fe_3_O_4_ and α-Fe_2_O_3_. An interconnected octahedral-shaped morphology was observed with a wide range of particle sizes ranging from 26 to 220 nm. The octahedral morphology was formed without using any surfactant which might be due to the usage of chloride as a starting material. Moreover, the presence of OH^−^ ions/high chemical potential can be attributed to provide the thermodynamically favorable conditions for the particle shape [33,34]. The attachments of carbon on the MNPs are clearly seen in Fig. 1B(b and c). The octahedral morphology was not changed for the sample calcined at 600 °C, whereas it was changed partially for the samples calcined at 800 °C. The fraction of carbon on the carbon-modified α-Fe_2_O_3_ was less compared to carbon-modified Fe_3_O_4_ which might be due to the higher temperature after heating. Fig. 1C shows the TEM micrograph of bare, carbon-modified Fe_3_O_4_ and α-Fe_2_O_3_. The octahedral-shaped particle looks like a cubic shape in different sizes. The morphology changes for the sample calcined at 800 °C is observed. The images were in correlation with the FESEM images. The morphology of the interconnected octahedral shape become partially spherical due to the high calcination temperature. The attachment of carbon materials on the MNPs and the fringes of MNPs are clearly seen from the HRTEM micrograph as shown in Fig. 1C(d-f). We have observed the higher carbon fraction in Fe_3_O_4_ compared to α-Fe_2_O_3,_ which supports the FESEM micrograph results. Furthermore, the prepared samples were undergone to VSM analysis for their magnetic characteristics. Fig. 1D shows the magnetic hysteresis loop of bare Fe_3_O_4_, Fe_3_O_4_/C, and α-Fe_2_O_3_/C. All the three samples exhibited ferromagnetic behavior. A higher saturation magnetization of 90 emu/g was observed for the bare Fe_3_O_4_ nanoparticles while it was reduced to 87 emu/g in case of carbon-modified Fe_3_O_4_ nanoparticles. The observed saturation magnetization value was nearly equal to the saturation magnetization value of bulk Fe_3_O_4_ [35]. The reduction in saturation magnetization value in the carbon-modified Fe_3_O_4_ can be attributed to the fraction of non-magnetic carbon attachment on the MNPs. The F3 sample of carbon-modified α-Fe_2_O_3_ showed drastic reduction in the saturation magnetization to 24 emu/g. This can be reasoned to the phase change of Fe_3_O_4_ to α-Fe_2_O_3_ due to higher calcination temperature and the non-magnetic fraction of carbon materials along with the MNPs. The coercivity of bare Fe_3_O_4_, Fe_3_O_4_/C, and α-Fe_2_O_3_/C was observed as 137, 139, and 251. The higher coercivity for α-Fe_2_O_3_/C compared to other two samples was reasoned to the phase change at higher calcination temperature.

**Fig. 2:**
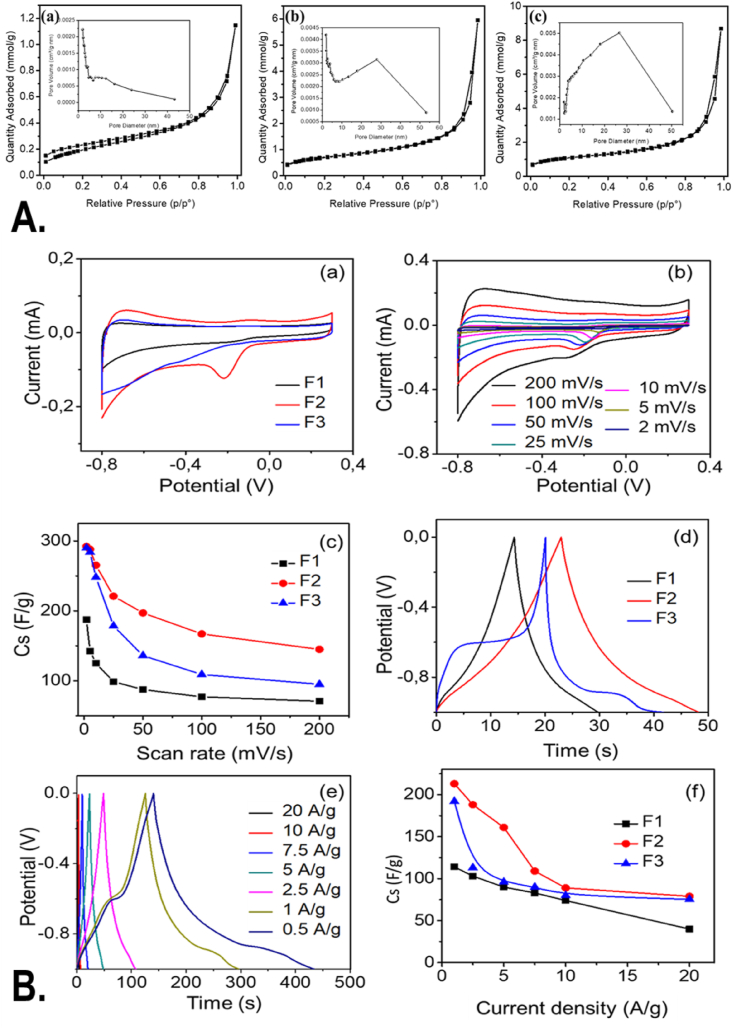
(A) N_2_ adsorption/desorption isotherm curve of (a) Fe_3_O_4_, (b) Fe_3_O_4_/C, and (c) α-Fe_2_O_3_/C. Inset shows pore size distribution. (B) (a) Comparative cyclic voltammogram of F1, F2, and F3 at 50 mV/s, (b) CV curve for F2 with different scan rate, (c) specific capacitance as a function of scan rate, (d) comparative GCD curve for F1, F2, and F3 at 5 A/g, (e) GCD curve for F2 at various current densities, and (f) specific capacitance of F1, F2, and F3 with different current densities. GCD, galvanostatic charge/discharge.

Furthermore, the surface area characteristics of the prepared samples were evaluated by the Brunauer–Emmett–Teller (BET) theory (BET) surface area analyzer. Fig. 2A shows N_2_ adsorption/desorption isotherm curve of (a) Fe_3_O_4_, (b) Fe_3_O_4_/C, and (c) α-Fe_2_O_3_/C. Inset shows the corresponding pore size distribution. The BET surface area of bare and carbon-modified Fe_3_O_4_ was measured as 16 and 56 m^2^/g. Bare Fe_3_O_4_ exhibited Type II isotherm which belongs to the microporous characteristics of the nanoparticles. The curve at P/P0 = 0.0–0.2 indicates the completion of monolayer and the initiation of multilayer adsorption. The carbon-modified Fe_3_O_4_ and α-Fe_2_O_3_ exhibited Type III isotherm shape with some area within the loop. The loop was similar to the H3-type hysteresis. As the loop did not show any limit or saturation at higher P/P0, H3-type hysteresis indicated to the aggregation of the particles. Carbon-modified α-Fe_2_O_3_ showed the highest surface area of 89 m^2^/g among the three samples. The average pore size of bare Fe_3_O_4_, carbon-modified Fe_3_O_4_, and carbon-modified α-Fe_2_O_3_ was observed as 6.1, 6.9, and 7.0 nm. The smaller pore size and the larger surface area of the prepared nanoparticles can be dedicated to different electrochemical and biocompatibility applications.

The cyclic voltammogram of Fe_3_O_4_, carbon-modified Fe_3_O_4_ and α-Fe_2_O_3_ with a scan rate of 50 mV/s is shown in Fig. 2B(a). It was seen that the shape of the curve was slightly distorted from the rectangle nature, which indicated the pseudo-capacitive nature along with double layer capacitive properties of the samples. The pseudo-capacitance process can be reasoned to the presence of iron oxide. The electric double layer capacitor process could be the result of the presence of carbon material in the metal oxides. It can also be noted that the pseudo-capacitive process was because of the result of oxidation and reductions between the ferrous and ferric ions through the intercalations with the electrolytes. The ferrous and ferric fraction within Fe_3_O_4_ was high compared to α-Fe_2_O_3,_ which could also be the reason for the larger integrated CV curve area for Fe_3_O_4_ compared to α-Fe_2_O_3._

The cyclic voltammogram for the carbon modified was measured with different scan rates from 2 to 200 mV/s as shown in Fig. 2B(b). The specific capacitance (*C*_*s*_) of the carbon decorated Fe_3_O_4_ and α-Fe_2_O_3_ electrode materials was calculated from the CV curves using the equation

(1) $$C_{s}=\frac{\int idV}{2m\times \Delta V\times v}$$

where *∫idV* is the integrated area of CV curves, *m* is the mass of electrode material on the electrode (g), Δ*V* is the potential window range, and *v* is the scan rate (V/s). The specific capacitance of the carbon decorated Fe_3_O_4_ and α-Fe_2_O_3_ was calculated as 292 and 290 F/g, which was higher than 198 F/g of bare Fe_3_O_4_. The enhanced capacitance value was observed around 54% for carbon-modified samples, which is the result of carbon addition within the composite materials. The linear decreasing order of specific capacitance rate was observed with increasing scan, which might be due to the internal resistance within the electrode, limiting the charge transfer process (Fig. 2B(c)).

Furthermore, galvanostatic charge/discharge studies are carried out to confirm the capacitive nature of Fe_3_O_4_ and its composites as shown in Fig. 2B(d) and Fig. S1. Fig. S1 shows the cyclic voltammogram (a and b) and galvanostatic charge/discharge curve (c and d) of bare Fe_3_O_4_ and carbon-modified α-Fe_2_O_3_. The galvanostatic charge/discharge (GCD) curve of carbon-modified α-Fe_2_O_3_ with different current densities ranging from 0.5 to 20 A/g was recorded and shown in Fig. 2B(e). The specific capacitance of these electrodes was determined using the following relation:

(2) $$C_{s}=\frac{I\times t}{m\times \Delta V}$$

where *I* (amp) is the applied current to the electrode, *t* is time taken for discharging (s), *m* is the mass of active electrode material (g), and Δ*V* is the potential window.

The calculated specific capacitance from the galvanostatic charge/discharge curves for bare Fe_3_O_4_, carbon-modified Fe_3_O_4_, and α-Fe_2_O_3_ is shown in Fig. 2B(f). The highest specific capacitance of 213 F/g was observed for carbon-modified Fe_3_O_4._ The carbon-modified α-Fe_2_O_3_ showed a specific capacitance of 192 F/g with a current density of 1 A/g, which was higher than that of bare Fe_3_O_4_ of 147 F/g. This enhancement may have originated from the higher BET-specific surface area and increased pore size of carbon-coated Fe_3_O_4_, which increases the electrolyte/electrode contact area and hence provides a greater number of active sites to store the charges. The larger value of specific capacitance with the modification carbon on Fe_3_O_4_ corroborated with the CV results. A decreasing trend in specific capacitance was observed with increasing current densities. At low current densities, a greater number of charges accumulated in the inner active sites of the electrode result in high specific capacitance values.

**Fig. 3:**
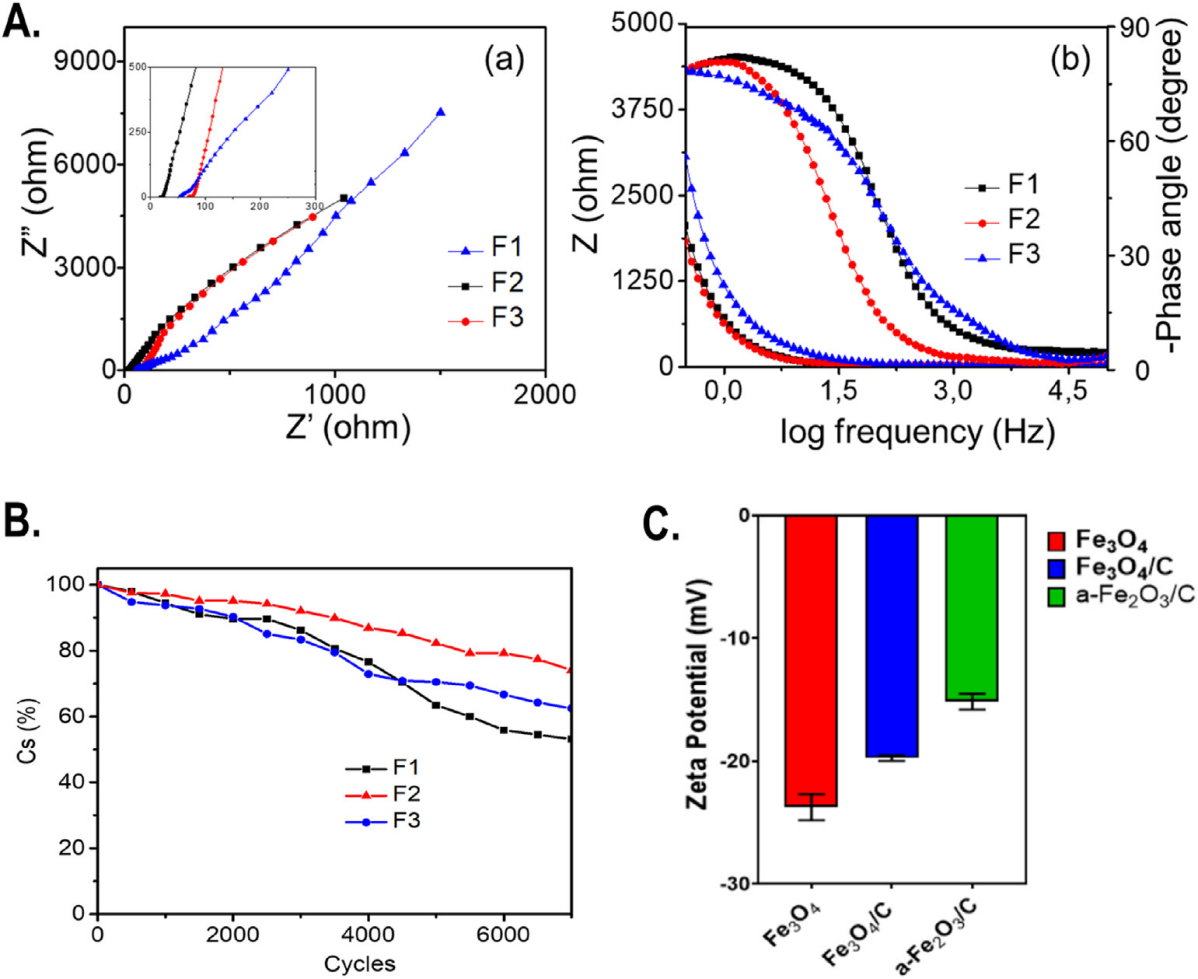
(A) (a) Nyquist plot and (b) Bode plot for Fe_3_O_4_ and its carbon supported nanocomposites. F1, F2, and F3 present Fe_3_O_4_, Fe_3_O_4_/C, and α-Fe_2_O_3_/C. (B) Cyclic stability of Fe_3_O_4_ (F1), Fe_3_O_4_/C (F2), and α-Fe_2_O_3_/C (F3). (C) Zeta potential of Fe_3_O_4_, Fe_3_O_4_/C, and α-Fe_2_O_3_/C.

Besides, EIS of Fe_3_O_4_ composite electrodes was carried out to verify our previous results and their corresponding Nyquist plots are illustrated in Fig. 3A(a) with an enlarged view (inset). At the high frequency region, there is a pseudo-semicircle in the Nyquist plot, which was reduced for carbon-coated Fe_3_O_4_. The data were evident to the fact that the introduction of carbon on Fe_3_O_4_ has significantly reduced the charge transfer resistance. Moreover, all three spectra showed straight line shape at low frequency, which is regarded as an important feature of a capacitor. Among the different spectra, F2 electrode showed the steeper line than other electrodes, the vertical shape of the curve confirmed its good capacitive performance.

In addition, the Bode plot in Fig. 3A(b) shows the impedance of the electrodes as a function of frequency. Generally, the impedance decreases as the frequency increases; however, the overall impedance value of carbon supported Fe_3_O_4_ showed slightly lower than bare Fe_3_O_4_, which is favorable for electron transport. Fig. 3A(b) shows the phase angle responses of Fe_3_O_4,_ carbon supported Fe_3_O_4_, and α-Fe_2_O_3_ samples for varying frequencies. The phase angle of all samples approach to zero at high frequency, but the phase angle of each electrode is distinguishable in the lower frequency region (less than 10 Hz). The phase angle of bare and carbon supported composites are very closer to −90°, which confirms the ideal capacitive nature of the prepared electrodes. Furthermore, to evaluate the cyclic stability of the electrode, all the samples were tested for 7,000 cycles at high current density of 5 A/g (Fig. 3B). All the samples showed 90% retention up to 2,000 cycles and slowly started decreasing with higher number of cycles. With 7,000 cycles, bare Fe_3_O_4,_ carbon-modified Fe_3_O_4_, and α-Fe_2_O_3_ showed 54%, 74%, and 63% of the capacitance retention, which confirms the enhancement in the stability of the electrode materials due to the addition of carbon material. To evaluate the alteration in zeta potential after carbon modification, zeta potential of all the samples was measured by zeta sizer in aqueous medium. As shown in Fig. 3C, zeta potential was found to be increased from −23.7 ± 0.7 mV of Fe_3_O_4_ to −19.7 ± 0.2 mV of Fe_3_O_4_/C and −15.1 ± 0.3 mV of α-Fe_2_O_3_.

**Fig. 4:**
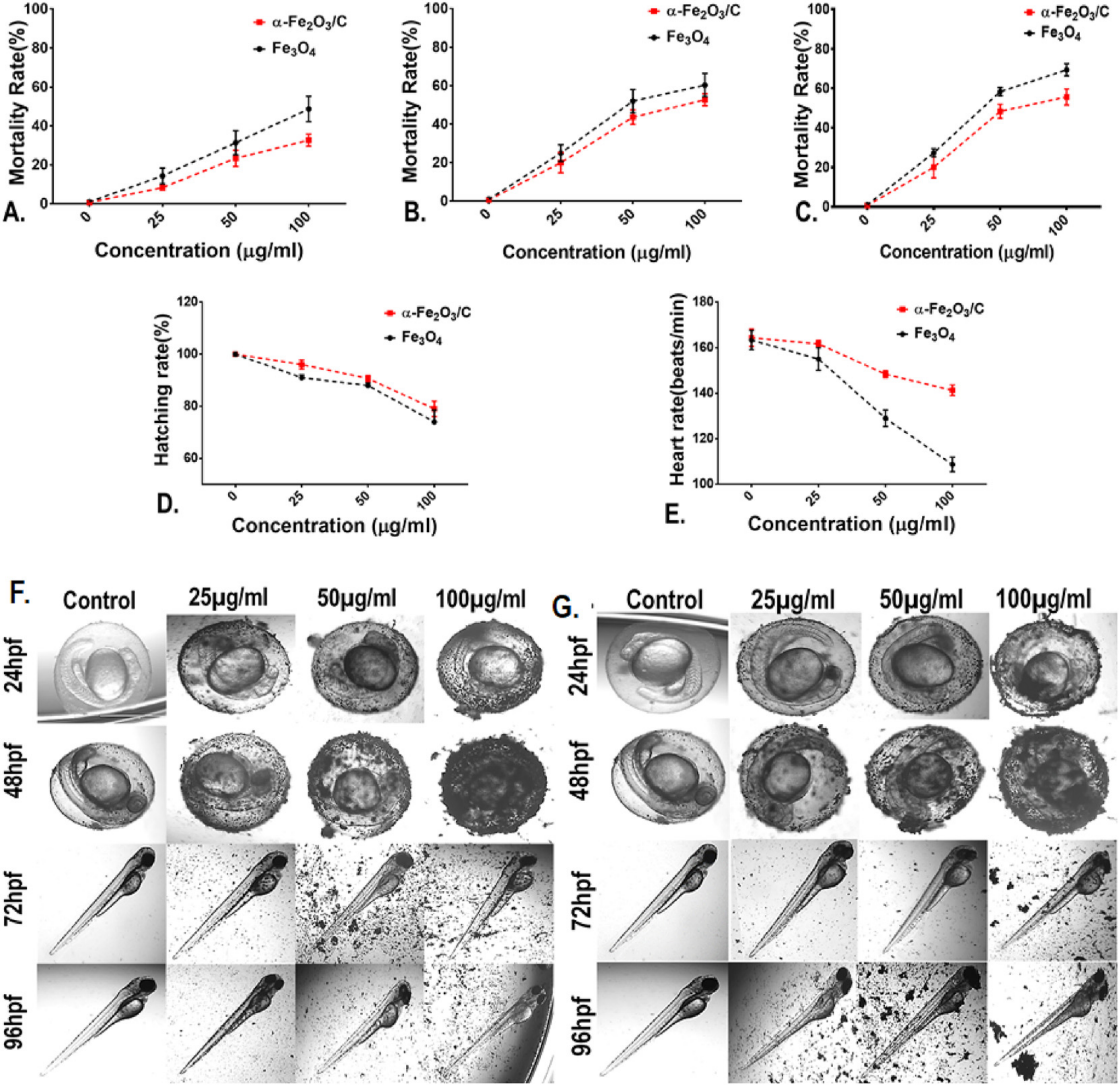
Assessment of different physiological parameters in zebrafish embryos exposed to Fe_3_O_4_ and α-Fe_2_O_3_/C. (A) Mortality rate at 24 hpf. (B) Mortality rate at 48 hpf. (C) Mortality rate at 72 hpf. (D) Hatching rate. (E) Heartbeat rate. Morphological analysis of zebrafish embryos exposed to (F) Fe_3_O_4_ and (G) α-Fe_2_O_3_/C nanoparticles. Attachment of nanoparticles to the chorion surface was clearly observed.

The physiochemical properties of Fe_3_O_4_, Fe_3_O_4_/C, and α-Fe_2_O_3_/C as determined by different techniques showed the significant alteration in their physicochemical properties. These alterations can be attributed to their utility as an electrochemical purpose. However, it was speculated to have a significant impact on their eco-biocompatibility. The hypothesis was verified by assessment of *in vitro* and *in vivo* biocompatibility of Fe_3_O_4_ and α-Fe_2_O_3_/C with HCT116 colon cell lines and embryonic zebrafish. The *in vitro* survivability analysis was performed with colon cell lines keeping in view of the fact that the nanoparticles show their effect on colon cells after passing though the alimentary canal. As shown in Fig. S2, MTT assay showed higher survivability of cells exposed to Fe_3_O_4_ in comparison to the cells exposed to α-Fe_2_O_3_/C indicating toward the higher biocompatibility of Fe_3_O_4_ in comparison to α-Fe_2_O_3_/C. The biocompatibility was further checked with embryonic zebrafish model for a detailed *in vivo* evaluation. To evaluate the cellular and morphological alteration due to exposure of Fe_3_O_4_ and α-Fe_2_O_3_/C to zebrafish embryos, different physiological parameters were checked. As shown in Fig. 4A–C, mortality rate was found to be reduced in zebrafish embryos exposed to α-Fe_2_O_3_/C compared to bare Fe_3_O_4_ nanoparticles at 24, 48, and 72 hpf. Hatching rate was also affected after exposure of carbon-modified Fe_3_O_4,_ which was higher compared to bare Fe_3_O_4_ (Fig. 4D). Moreover, assessment of heartbeat rate also showed a significant inclination in embryos exposed to carbon-modified Fe_3_O_4_ than bare Fe_3_O_4_ (Fig. 4E). Alteration in these parameters can be attributed to change in BET surface area of Fe_3_O_4_ as determined experimentally.

It can be argued that due to higher surface area, the carbon-modified MNPs attach to the chorion surface of embryos and get less internalized compared to the non-modified ones [36]. Moreover, due to higher porous nature of α-Fe_2_O_3_/C compared to Fe_3_O_4_, there has been less interaction with the protein enzymes present at the chorion responsible for hatching leading to higher hatching rate [37]. The interesting fact of less heartbeat rate can be intrigued toward the lower magnetic nature of α-Fe_2_O_3_/C due to carbon modification. To verify the fact, the morphology assessment of embryos exposed to Fe_3_O_4_ and α-Fe_2_O_3_/C was done using microscopy. As shown in Fig. 4F and G, it was clearly found that the Fe_3_O_4_ nanoparticles were accumulated at the surface of chorion at a higher rate. Interestingly, there was no notochord development abnormalities while the pericardial edema was significant in case of Fe_3_O_4_ nanoparticle exposure.

**Fig. 5:**
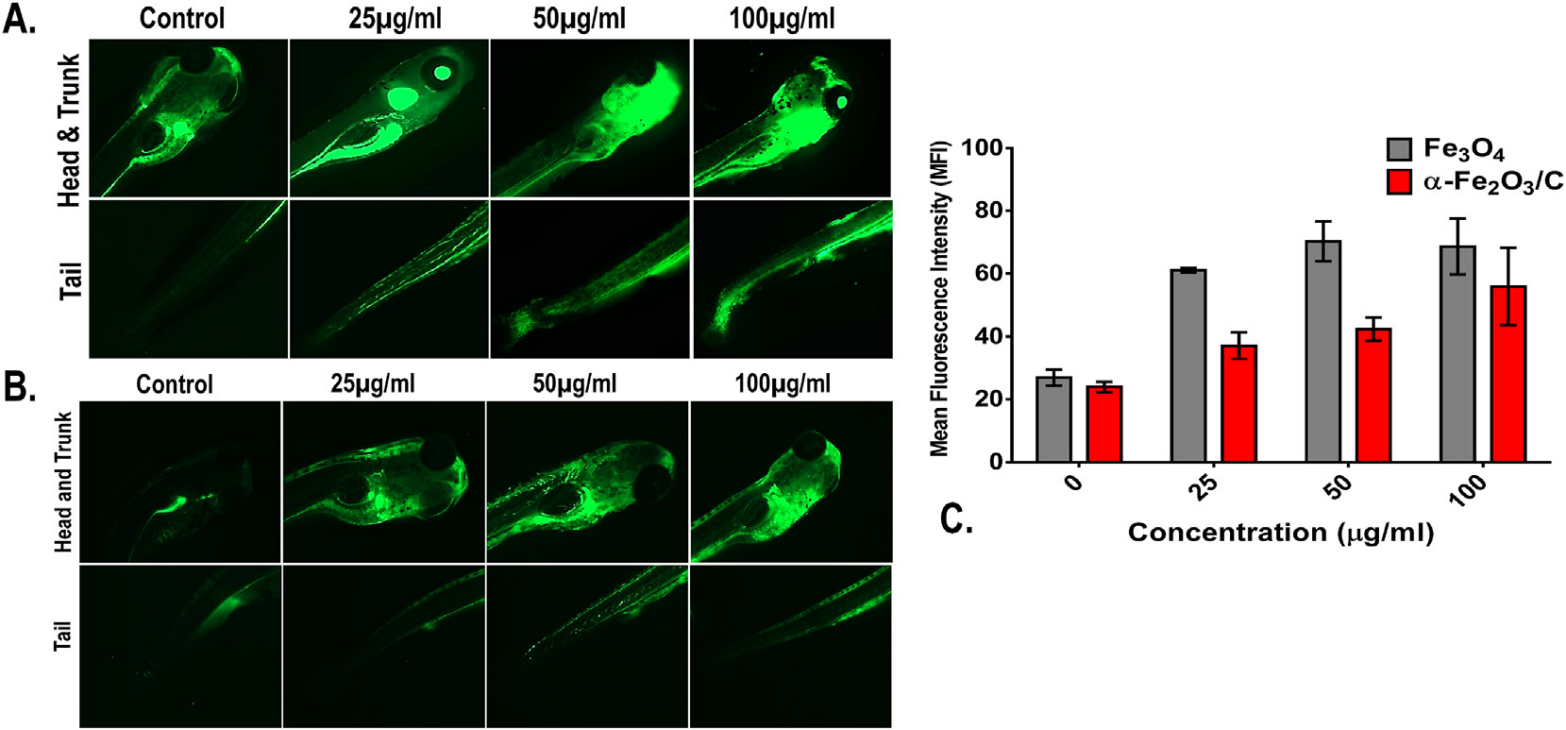
Oxidative stress analysis of zebrafish embryos exposed to (A) Fe_3_O_4_ and (B) α-Fe_2_O_3_/C nanoparticles. (C) Bar graph presenting the mean fluorescent analysis of the DCFDA dye used for staining the embryos for determination of oxidative stress.DCFDA, 2′,7′-dichlorofluorescin diacetate.

**Fig. 6:**
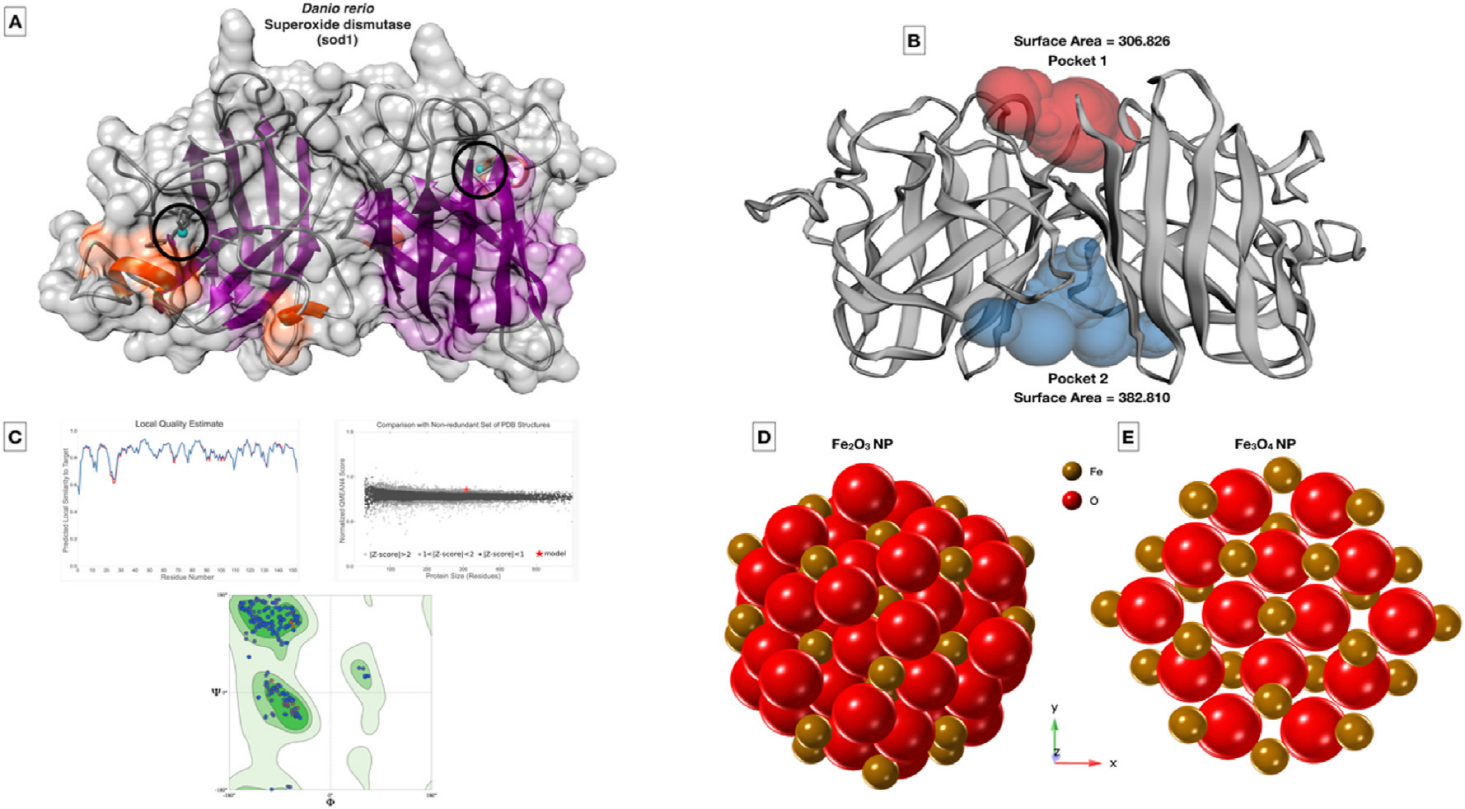
(A) Molecular modeling of superoxide dismutase (sod1) protein from *Danio rerio* obtained from SWISS-Model server. (B) Active site pockets depicted as Pocket 1 and 2 obtained from CastP server. (C) Quality assessment of the obtained homology model from Swiss-Model server. (D) Structure of α-Fe_2_O_3_ nanoparticle modeled by Crystal Maker software. (E) Structure of α-Fe_3_O_4_ nanoparticle modeled by Crystal Maker software. The aforementioned *in silico* analysis has been depicted only with sod1 which is responsible for ROS generation.

**Fig. 7:**
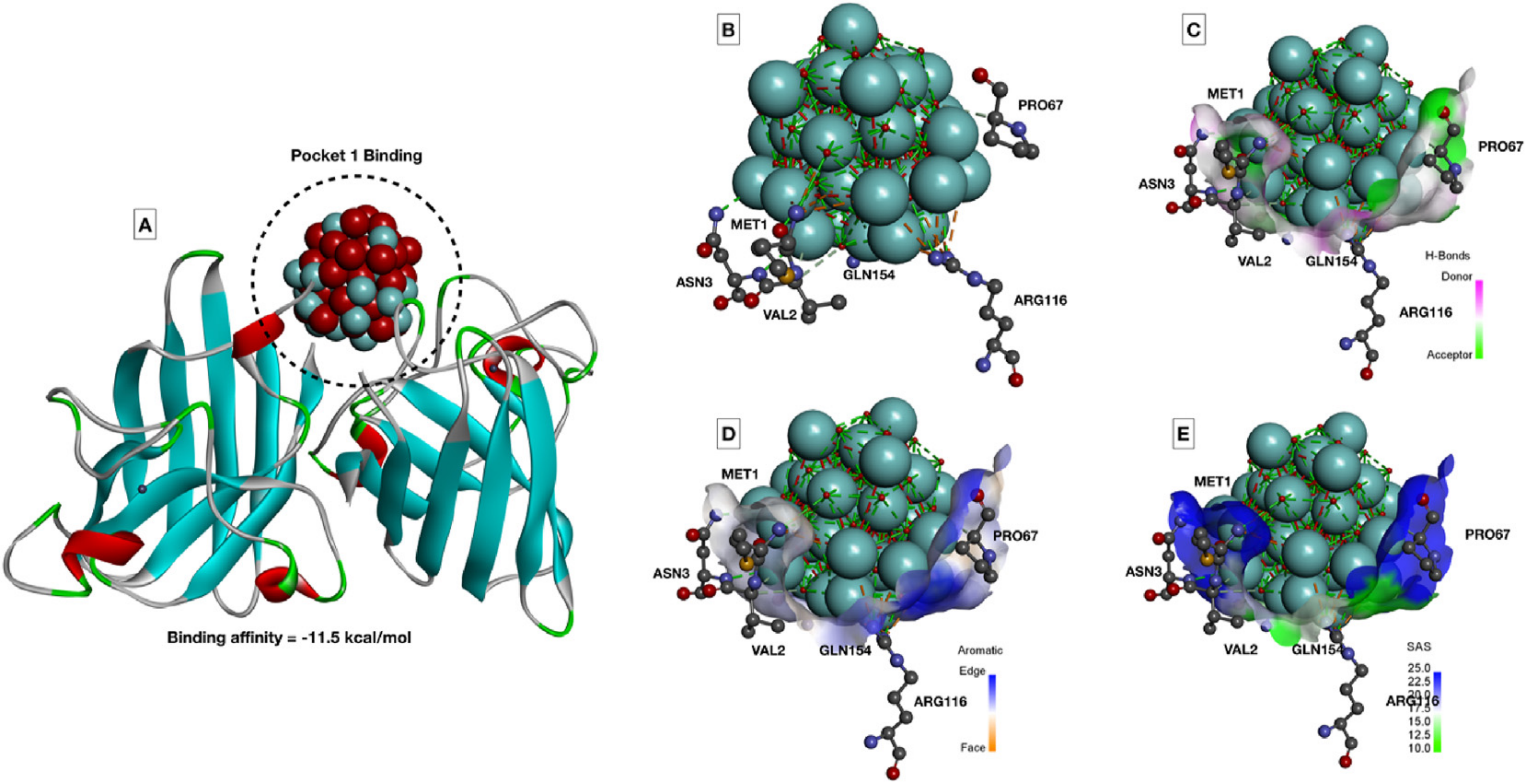
Molecular docking of α-Fe_2_O_3_ nanoparticle with sod1 protein. (A) Binding orientation of α-Fe_2_O_3_ nanoparticle with binding affinity of −11.5 kcal/mol docked in the active site Pocket 1. (B) Interacting amino acid residues of sod1 with α-Fe_2_O_3_ nanoparticle. (C–E) Hydrogen bonding, aromaticity, and solvent accessible surface area of interacting residues with α-Fe_2_O_3_ nanoparticle.

**Fig. 8:**
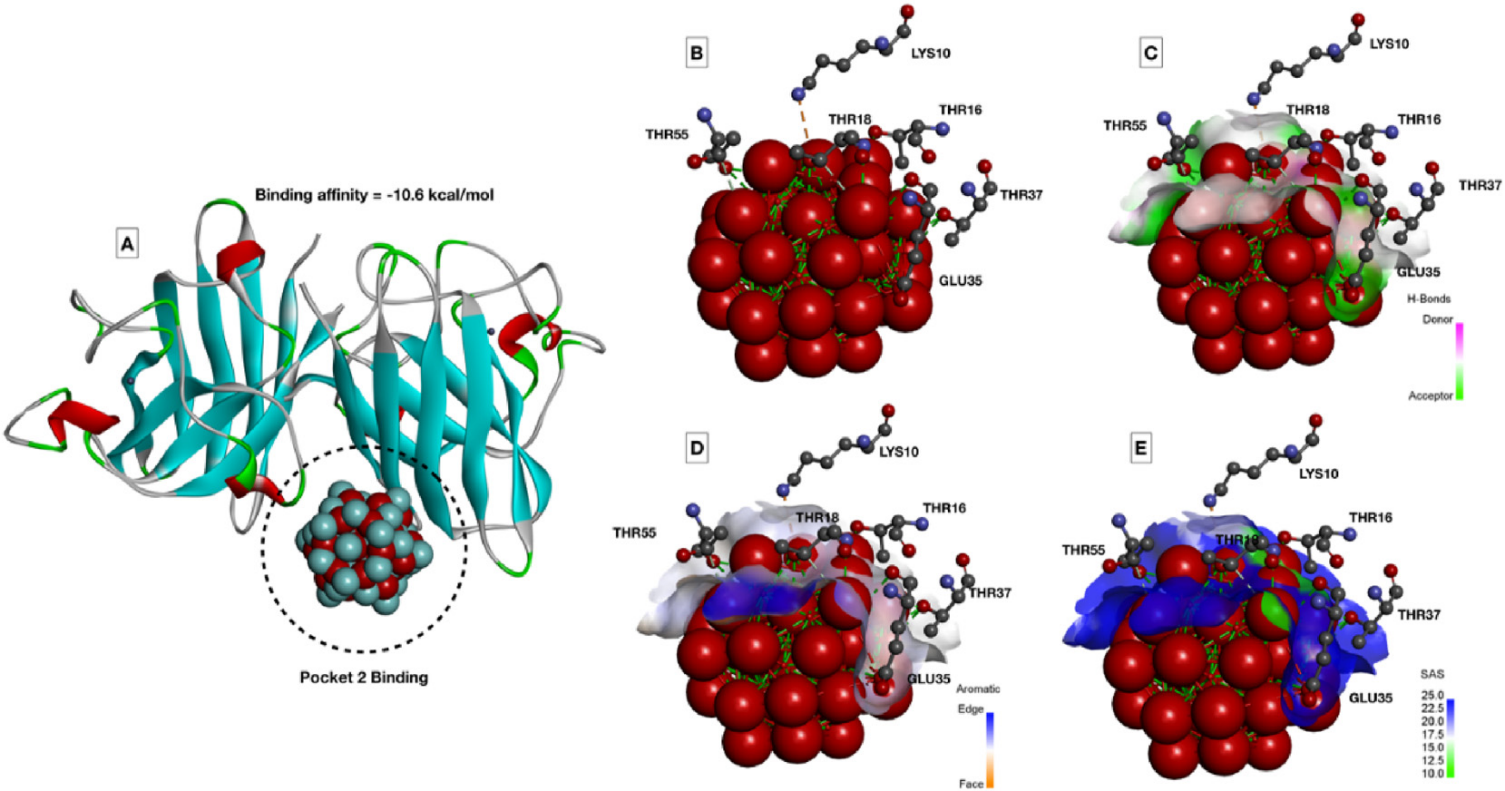
Molecular docking of α-Fe_3_O_4_ nanoparticle with sod1 protein. (A0 Binding orientation of α-Fe_2_O_3_ nanoparticle with binding affinity of −10.6 kcal/mol docked in the active site Pocket 2. (B) Interacting amino acid residues of sod1 with α-Fe_3_O_4_ nanoparticle. (C–E) Hydrogen bonding, aromaticity, and solvent accessible surface area of interacting residues with α-Fe_3_O_4_ nanoparticle.

The observation can be attributed toward the lower internalization of α-Fe_2_O_3_/C compared to Fe_3_O_4_ due to less surface area. The results indicated toward the fact that accumulation of the nanoparticles at the surface area and skin surface of the 72 hpf embryos could lead toward the hypoxic condition that can alter the balance of oxidative stress [36]. Hence, to verify the fact, oxidative stress analysis was performed using 2′,7′-dichlorofluorescin diacetate (DCFDA) staining to the Fe_3_O_4_ and α-Fe_2_O_3_/C exposed embryos and the mean fluorescent intensity was calculated to compare the oxidative stress induction. As shown in Fig. 5, dose-dependent induction of reactive oxygen species (ROS) was found in both the case of Fe_3_O_4_ and α-Fe_2_O_3_/C nanoparticle exposure. The ROS intensity was found to be increased with increase in exposure concentration; however, there was significant reduction in the fluorescent intensity in case of α-Fe_2_O_3_/C nanoparticle compared to bare Fe_3_O_4_ nanoparticles. Discrepancy in oxidative stress due to nanoparticle interference has been acknowledged to their influential interaction with oxidative stress regulatory proteins like sod1. It was argued that differential Fe_3_O_4_ and α-Fe_2_O_3_/C nanoparticle interaction was playing a crucial role in the determination of their ROS induction phenomenon [38]. The fact was verified by the messenger ribonucleic acid (mRNA) expression of untreated and treated embryos by RT-PCR. As shown in Fig. S3, a significant increase in fold change expression of sod1 was found with increasing concentration of exposure of both nanoparticles; however, there was a clear significant increase in mean fold change expression in case of Fe_3_O_4_ nanoparticle exposure compared to α-Fe_2_O_3_/C nanoparticles. Furthermore, the effect of differential Fe_3_O_4_ and α-Fe_2_O_3_/C nanoparticle interaction on sod1 was studied by *in silico* approach. As shown in Fig. 6, the molecular structure of sod1 protein exhibited the presence of firm active pocket for interaction of determined molecular structure of Fe_3_O_4_ and α-Fe_2_O_3_/C nanoparticles. As shown in Fig. 7, docking analysis exhibited a firm interaction of α-Fe_2_O_3_ nanoparticle with sod1 via arginine (Arg), proline (pro), methionine (Met), glycine (Gly), and valine (Val) through hydrogen bonding with binding affinity of −11.5 kcal/mol; these interactions can be attributed to the influential change in structural and functional integrity of sod1 [39]. The variation in sod1 interaction with α-Fe_3_O_4_ nanoparticle can be seen in Fig. 8, where the nanoparticles were found to be interacting through different amino acids like lysine (Lys), threonine (Thr), and glutamine (Glu) with binding affinity of −10.6 kcal/mol.

**Fig. 9:**
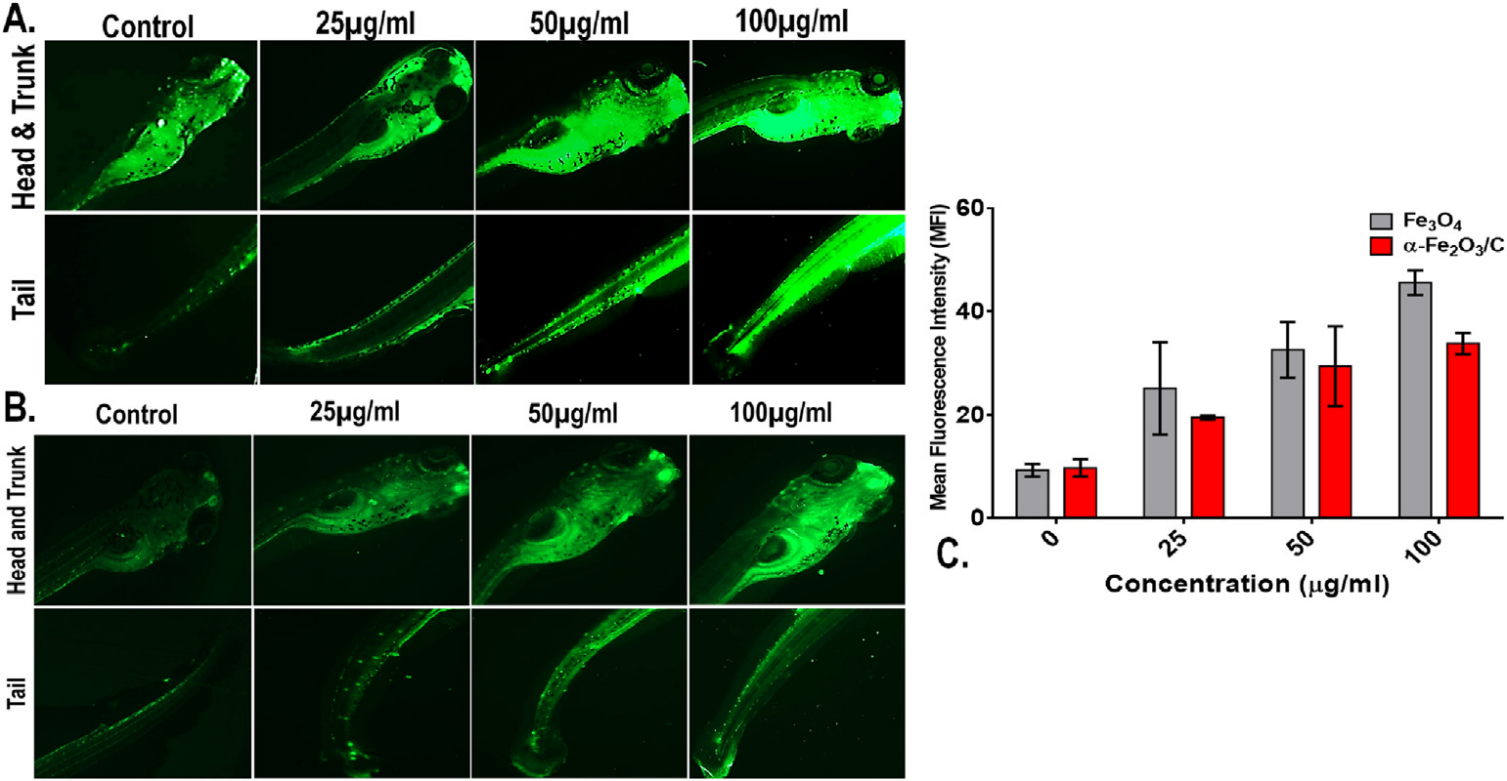
Apoptosis analysis of zebrafish embryos exposed to (A) Fe_3_O_4_ and (B) α-Fe_2_O_3_/C nanoparticles. (C) Bar graph presenting the mean fluorescent analysis of the Acridine orange dye used for staining the embryos for determination of Apoptosis.

Dysregulation of oxidative stress has been reported to be one of the major cause of cellular physiology abnormalities and enhancement in cell death activities like apoptosis [40]. Hence, it was hypothesized that the reduced oxidative stress in embryos exposed to α-Fe_2_O_3_/C nanoparticles compared to Fe_3_O_4_ nanoparticles will lead toward less apoptosis. The hypothesis was experimentally verified by Acridine orange staining, which has been established as a known marker dye to determine the cellular apoptosis in cells [41,42]. As shown in Fig. 9, it was found that the Acridine orange intensity was increasing with the increase in concentration; however, there was reduction of intensity in α-Fe_2_O_3_/C nanoparticles exposed to embryos. The data were in correlation with our assumption and evident to the verification of hypothesized fact. Similar observation has been found by different literature in case of exposure to other metallic oxide nanoparticles like ZnO, CuO, and TiO_2_ nanoparticles [36,43,44]. The observed data and results were also paving the pathway for mechanism deduction in the biocompatibility. Hence, with reference to previous literatures [45] and our experimental analysis, the mechanism of the enhanced *in vivo* biocompatibility of α-Fe_2_O_3_/C nanoparticles compared to bare Fe_3_O_4_ nanoparticles can be deduced as follows: carbon modification of Fe_3_O_4_ nanoparticles induce higher surface area with higher porosity and less magnetization saturation. The alteration of these parameters causes less attachment and internalization of α-Fe_2_O_3_/C nanoparticles at the surface and inside the embryos. Hence, the induction of hypoxic condition is comparatively less leading to less generation of ROS. Moreover, due to less internalization that occurred as a result of less magnetization value, the interaction of α-Fe_2_O_3_/C nanoparticles would have been less with protein molecules inside the cells which are responsible for cellular physiology like oxidative stress and apoptosis. The combinatorial effect of these two phenomena leads toward the less oxidative stress and less apoptosis in case of α-Fe_2_O_3_/C nanoparticle exposure compared to Fe_3_O_4_ nanoparticles.

Hence, the detailed study evident toward the higher utility of synthesized carbon-modified Fe_3_O_4_ nanoparticles with novel method and indicated toward the higher biocompatibility.

## 4 Conclusion

We successfully synthesized novel octahedral-shaped morphology of Fe_3_O_4_ by simple chemical oxidation method. The chemically prepared MNPs were surface modified with carbon by chemical pyrolysis process. The single phase of bare Fe_3_O_4_ and phase change due to higher calcination temperature was confirmed from XRD analysis. The octahedral-shaped morphology and the particle size distribution were analyzed by FESEM, TEM, and HRTEM microscopic measurements. Bare and carbon-modified Fe_3_O_4_ showed the highest saturation magnetization of 90 and 82 emu/g. α-Fe_2_O_3_/C sample exhibited the lesser saturation magnetization of 24 emu/g. The reduction in the saturation magnetization is due to the non-magnetic fraction of carbon materials and the phase transformation occurred at higher calcination temperature. The capacitive behavior of the prepared samples was analyzed by cyclic voltammogram and galvanostatic charge/discharge studies. The highest specific capacitance of 213 F/g was observed for carbon-modified Fe_3_O_4_ and carbon-modified α-Fe_2_O_3_ showed a specific capacitance of 192 F/g. The electrochemical studies also confirmed that incorporation of carbon with Fe_3_O_4_ significantly reduced the charge transfer resistance and increased the capacitive nature. Cyclic performance was tested up to 7,000 cycles. *In vivo* biocompatibility analysis showed reduction in toxicity of Fe_3_O_4_ nanoparticles by carbon modification by inducing less morphological abnormalities, oxidative stress, and apoptosis in zebrafish embryos. The higher magnetization, superior electrochemical characteristics, and higher biocompatibility suggested that the carbon-modified Fe_3_O_4_ would be the best among other two materials which are suitable for the negative electrode materials for supercapacitor applications and will be also ecofriendly with respect to environmental and clinical aspects.

## CRediT author contribution statement

**Suresh K. Verma:** Conceptualization, Methodology, Software, Validation, Formal analysis, Investigation, Resources, Data curation, Writing – original draft, Writing – review & editing, Visualization, Supervision, Project administration, Funding acquisition. **Arun Thirumurugan:** Conceptualization, Methodology, Software, Validation, Formal analysis, Investigation, Resources, Data curation, Writing – original draft, Writing – review & editing, Visualization. **Pritam Kumar Panda:** Conceptualization, Investigation, Software, Methodology. **Paritosh Patel:** Methodology, Formal analysis, Investigation, Resources. **Aditya Nandi:** Methodology, Investigation, Formal analysis. **Ealisha Jha:** Methodology, Formal analysis, Investigation, Resources. **K. Prabakaran:** Methodology, Formal analysis, Investigation, Resources. **R. Udayabhaskar:** Methodology, Formal analysis, Investigation, Resources. **R.V. Mangalaraja:** Methodology, Formal analysis, Investigation, Resources. **Yogendra Kumar Mishra:** Methodology, Formal analysis, Investigation, Writing – review & editing, Resources, Formal analysis. **Ali Akbari-Fakhrabadi:** Methodology, Formal analysis, Investigation Writing – review & editing, Resources. **Mauricio J. Morel:** Methodology, Formal analysis, Investigation Writing – review & editing, Resources. **Mrutyunjay Suar:** Conceptualization, Methodology, Software, Validation, Resources, Data curation, Writing – review & editing, Supervision, Project administration, Funding acquisition. **Rajeev Ahuja:** Conceptualization, Methodology, Software, Validation, Resources, Data curation, Writing – review & editing, Supervision, Project administration, Funding acquisition.

## Declaration of competing interest

The authors declare that they have no known competing financial interests or personal relationships that could have appeared to influence the work reported in this paper.
