# Supplementary material for: Altered electrochemical properties of iron oxide nanoparticles by carbon enhance molecular biocompatibility through discrepant atomic interaction
Source: Mater Today Bio. 2021 Sep 4;12:100131. doi: 10.1016/j.mtbio.2021.100131 (PMC8479829; doi:10.1016/j.mtbio.2021.100131)
Supplement: Multimedia component 1 [file mmc1.docx]

**Supplementary Information**

**Altered electrochemical properties of Iron oxide nanoparticles by carbon enhance molecular biocompatibility through discrepant atomic interaction**

Suresh K Verma^1‡^**^§^***, Arun Thirumurugan^1Ɏ^*, Pritam Kumar Panda^§^, Paritosh Patel^‡^, Aditya Nandi^‡^, Ealisha Jha^‡^, K. Prabakaran^$^, R. Udayabhaskar^Ɏ^, R.V. Mangalaraja **^£,^**^¶^, Yogendra Kumar Mishra^€^, Ali Akbari-Fakhrabadi^†^, Mauricio J. Morel^Ɏ^, Mrutyunjay Suar^‡*^, Rajeev Ahuja^§,^**^¥*^**

**^‡^**School of Biotechnology, KIIT University, Bhubaneswar, India, 751024

**^§^**Condensed Matter Theory Group, Materials Theory Division, Department of Physics and Astronomy, Uppsala University, Box 516, SE-75120, Uppsala, Sweden

**^†^**Advanced Materials Laboratory, Department of Mechanical Engineering, University of Chile, Santiago, Chile.

**^$^**SRM Research Institute, SRM Institute of Science and Technology, Kattankulathur, Chennai, Tamil Nadu, India – 603203^.^

**^£^**Advanced Ceramics and Nanotechnology Laboratory, Department of Materials Engineering, Faculty of Engineering, University of Concepción, Concepción, 4070409, Chile.

**^¶^**Technological Development Unit (UDT), University of Concepcion, Coronel Industrial Park, Coronel, Chile

**^€^**Mads Clausen Institute, NanoSYD, University of Southern Denmark, Alsion 2, Denmark

**^¥^**Applied Materials Physics, Department of Materials and Engineering, Royal Institute of Technology (KTH), SE-10044, Stockholm Sweden

^Ɏ^Instituto de Investigaciónes Científicas y Tecnológicas (IDICTEC), Universidad de Atacama, Copayapu 485, Copiapó, Chile.

^1^ Authors with equal contribution as first author.

*Corresponding author: [suresh.verma@physics.uu.se](mailto:suresh.verma@physics.uu.se), [arunthiruvbm@gmail.com](mailto:arunthiruvbm@gmail.com), [msbiotek@yahoo.com](mailto:msbiotek@yahoo.com), [rajeev.ahuja@physics.uu.se](mailto:rajeev.ahuja@physics.uu.se) .

**Materials and Methods**

**Cell culture and MTT assay**

Colon cancer cell line (HCT116) was procured from National Centre for Cell Sciences (NCCS) Pune, India. The maintenance of the cells was done in complete culture medium consisting of Dulbecco’s modified Eagle’s medium (DMEM) supplemented with 10% heat-inactivated foetal bovine serum (Himedia), L-glutamine 2mM, penicillin 100IU/ml and streptomycin 100µg/ml and gentamicin 0.5% at 37^0^C in a saturated humidity atmosphere with 5% CO2. For MTT assay, 2.5х10^4^ Cells per well were seeded in 100µl of complete medium and incubated for 24h. After proper adherence, the medium was changed by 200µl cell medium with Fe_3_O_4_ and α-Fe_2_O_3_/C made to final concentration of a range of 10-500 µg/ml. The treated cells were then incubated for 24h humidified atmosphere with 5% CO_2_ at 37^0^C. Followed by incubation, washing of the cells was done twice with PBS (pH7.4) and MTT solution was added for 3 hours. The formazen crystals formed were then dissolved in crystal dissolving buffer (11gm SDS in 50ml of 0.02M HCl and 50ml isopropanol). The absorbance was taken at 570 nm in ELISA plate reader (Epoch, Biotek, Germany). The amount of color product corresponds to the number of viable cells. Non-treated cells absorbance was taken as a reference for calculating 100% cellular viability. LC50 was determined by determining the concentration of NPs at 50% viability of cells.

**
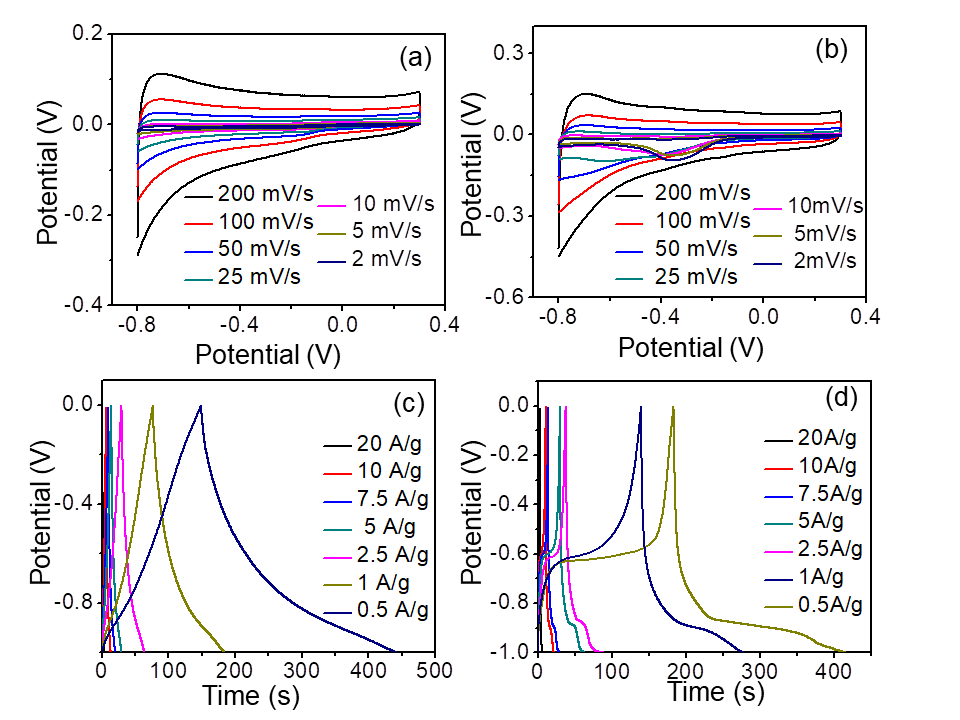
**

**Figure. S1.** CV (a&b) and GCD curve (c&d) curve bare Fe_3_O_4_ and carbon modified α-Fe_2_O_3_


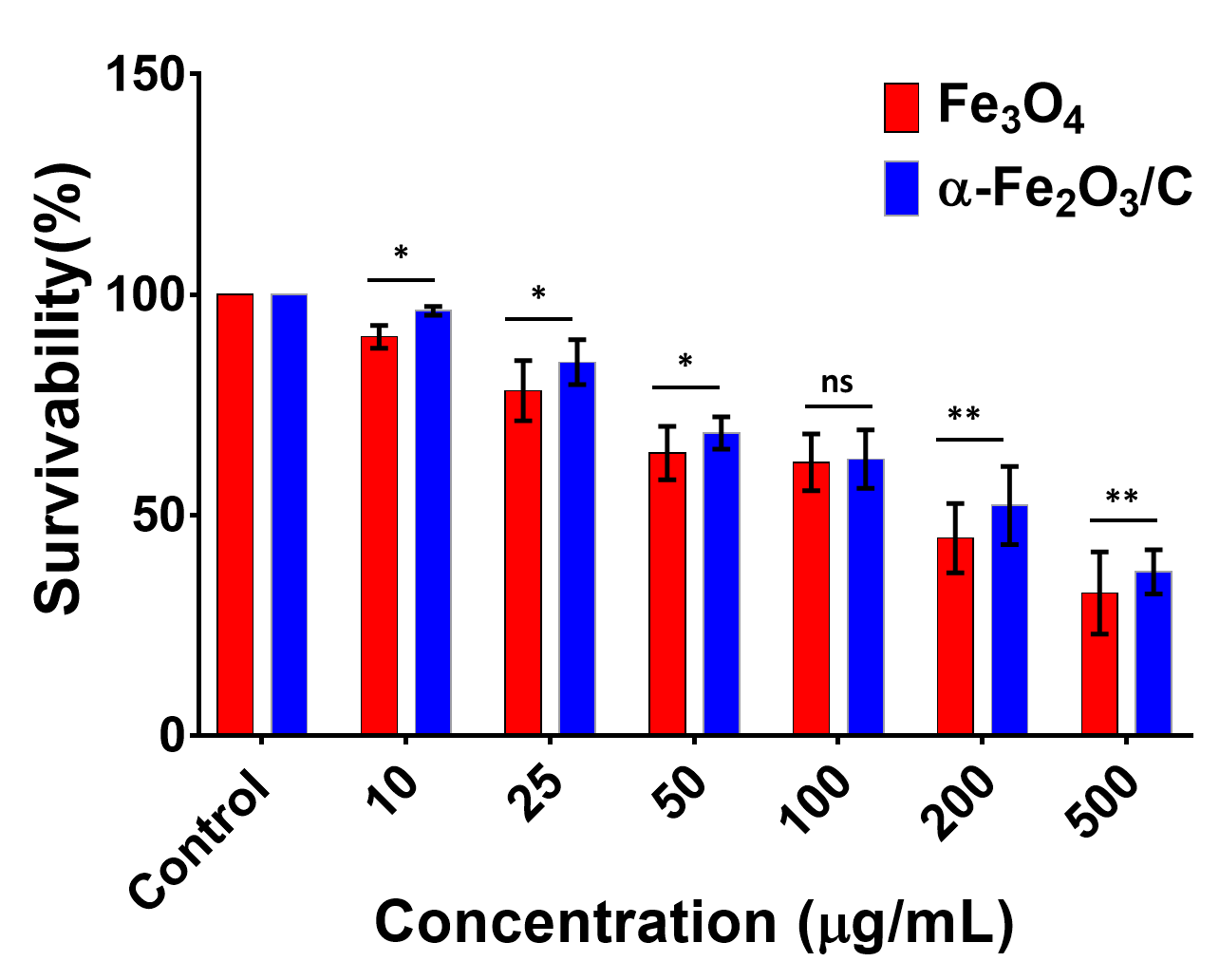


**Figure. S2.** Survivability of HCT116 cells exposed to Fe_3_O_4_ and carbon modified α-Fe_2_O_3_ as determined by MTT assay_._ The values represent the mean ± SD of three independent experiments. **P >0.1, ***P >0.01, ****P >0.001 denotes compared significant change at each exposed concentration as obtained from post hoc analysis after one-way ANOVA


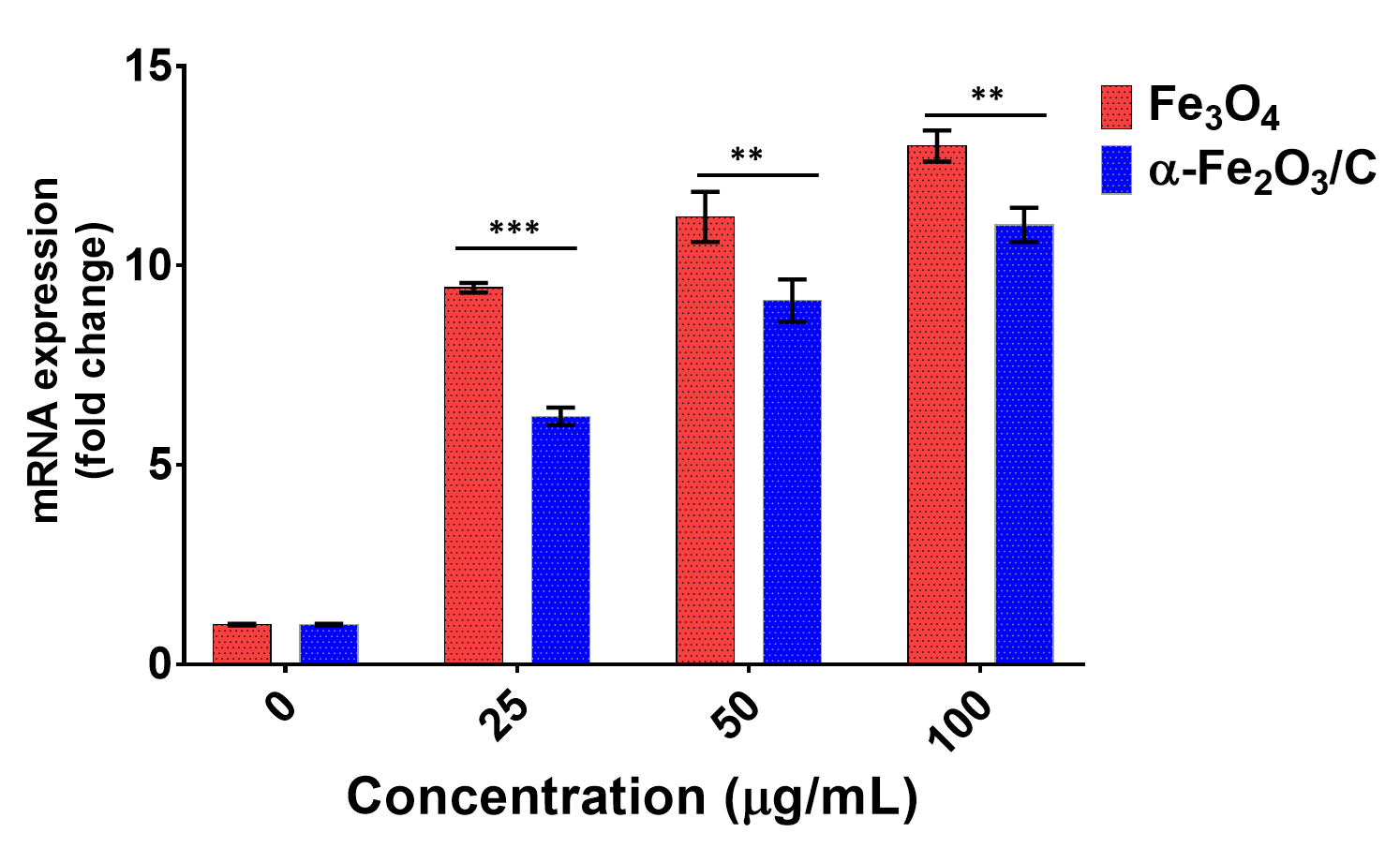


**Figure. S3.** Fold change expression of mRNA expression of Sod1 in zebrafish embryos cells exposed to Fe_3_O_4_ and α-Fe_2_O_3_/C nanoparticles for 72hrs as determined by RT-PCR analysis. The values represent the mean ± SD of three independent experiments. **P >0.1, ***P >0.01, ****P >0.001 denotes compared significant change at each exposed concentration as obtained from post hoc analysis after one-way ANOVA
